# Detection, Inspection, Return: An Object-Based Classification and Metric of Fixations in Complex Scenes

**DOI:** 10.1162/OPMI.a.319

**Published:** 2026-01-15

**Authors:** Marcel Linka, Benjamin de Haas

**Affiliations:** Experimental Psychology, Justus Liebig University Giessen, Giessen, Germany

**Keywords:** scene viewing, fixations, free viewing, eye movements

## Abstract

Analyses of human gaze behaviour towards complex scenes typically aim to explain heatmaps or scan-paths. While heatmaps lack temporal information, scan-paths aim for a level of detail which often is impractical. We introduce a novel approach, based on the premise that most fixations target objects and do so in meaningfully different ways, depending on temporal context: Detection fixations (D) foveate an object for the first time; Inspection fixations (I) successively target object details; and Return fixations (R) revisit a previously fixated object after going elsewhere. To test the hypothesis that these classes capture distinct fixation profiles, we reanalysed a large dataset of scene fixations. We computed separate heatmaps for D, I, and R and found significantly higher inter-observer consistency within than between classes. Across fixations landing on different semantic features, the proportion of D, I, and R fixations varied consistently, and a semantic salience model trained to predict each type of fixations independently learned diverging distributions of feature weights. Further, we found a shift from D to I and R across viewing time, in line with previous findings on ambient and focal viewing modes. We tested and confirmed that the dynamics of this shift varied as a function of trial duration. Finally, we highlight the recent application of the D, I, R classification as a metric for gaze comparisons in the context of dynamic scenes, in which scan-path similarity metrics fail. We propose the D, I, and R classification as a computationally simple yet powerful tool to classify spatiotemporal aspects of scene fixations in an object-based and intuitive manner and provide well-documented code to implement it. Future research may explore potential functional differences between D, I, and R fixations.

## INTRODUCTION

Our ability to resolve the detail and clutter of visual scenes is best at the fovea and quickly falls off outside of that (Rosenholtz, 2016). This is one reason we constantly move our eyes. At each moment in time, the oculomotor system must decide when and where to look next. These decisions crucially determine what visual information we process when and at which level of detail.

Research examining these decisions focuses on two attentional mechanisms: Stimulus features, which guide attention bottom-up, and top-down mechanisms, which are observer- and goal-specific (Borji & Itti, 2013).

To investigate these mechanisms in free-viewing behaviour towards complex scenes, typical approaches aim to explain the spatial distribution of fixations in the form of heatmaps, or their exact sequence in the form of scan-paths. Heatmaps and scan-paths typically are pixel-based and agnostic to visual objects and their features. While heatmaps discard temporal information altogether, scan-paths aim for a level of detail which is impractical and may not be relevant for many research questions and applications. Inspired by several streams of research, we introduce and test a novel way of analyzing fixations in an object-based manner that takes into account both spatial and temporal aspects of gaze.

### Fixations in Natural Viewing Behaviour

Salience models of complex scenes traditionally focused on low-level image features (e.g., the local contrast in orientation, intensity and colour; Harel et al., 2007; Itti & Koch, 2000), while more recent models include high-level object features such as faces and text, which are either explicitly encoded (Xu et al., 2014) or learned as ‘deep features’ by artificial neural networks (Kümmerer et al., 2016). Importantly, when considering such high-level object features for predicting natural gaze behaviour, they outweigh low-level features (Xu et al., 2014). Other models take local meaning (Henderson & Hayes, 2017) or elements of scene context into account (e.g., scene gist and object-context congruency; Borji & Itti, 2013; Bornstein et al., 2011; Hayes & Henderson, 2021; Murlidaran & Eckstein, 2025; Torralba et al., 2006). Such models are typically trained and evaluated using human free viewing data.

A separate body of research approaches visual attention towards scenes from a top-down perspective and studies the effects of distinct tasks, goals and instructions (Borji & Itti, 2013; Yarbus, 1965). Research on eye movements during naturalistic visuo-motor tasks such as driving (Land & Lee, 1994) or sandwich making (Hayhoe et al., 2003) has shown that certain fixation sequences towards task-relevant objects, referred to as visual routines, can be highly functional and task-specific (Hayhoe, 2000; Ullman, 1984). Further, studies examining eye movements during reading have identified distinct classes of functional fixations—such as first fixations (the initial fixation on a word), refixations (subsequent fixations on the same word), and regressions (saccades against the orthographic direction, to a previous word in the text)—that likewise reflect task demands and processing strategies (Engbert et al., 2002; Vitu & McConkie, 2000).

As for free viewing behaviour, typical models describe and predict the spatial distribution of fixations for a given scene in a time-pooled manner, e.g., using heatmaps. An advantage of this approach is that it is easy to compute and interpret. However, it does not take into account the spatiotemporal dynamics of gaze behaviour. This is in contrast to scan-path models, which commonly aim to predict the exact sequence of fixations, e.g., by using biologically and statistically inspired algorithms (Kümmerer & Bethge, 2021; Xia et al., 2019; Zanca et al., 2020), even addressing parameters of individual differences in different task settings (Schwetlick et al., 2023). Although some of these models achieve significant predictive performance, there are no intuitively interpretable metrics that capture both spatial and temporal aspects of these models. At the same time, scan-path similarity metrics often are based on coarse spatial grids rather than pixels to avoid combinatorial explosion (Cristino et al., 2010), suffer from a lack of intuitive interpretability and inconsistency (Kümmerer & Bethge, 2023) and are not applicable to dynamic stimuli, for which the precise timing of gaze shifts matter and which can evoke pursuit events (Roth et al., 2023). Object-based classifications address these limitations by inherently reducing the dimensionality of gaze traces and tracking the time-varying spatial locations of objects (Mengers et al., 2025; Roth et al., 2022, 2023, 2024).

The idea that fixations are object-driven and may be classified according to temporal order goes back to the earliest work on eye movements (Buswell, 1935; Mannan et al., 1997; Yarbus, 1965). Buswell (1935) studied subjects freely viewing images and concluded that fixations tend to be drawn to visual objects, particularly depicted people. He also identified two types of fixations; relatively short ones, which sample the main aspects of the image, and longer ones, that are concentrated over a smaller region. Later research then confirmed these findings, demonstrating a systematic increase in fixation durations and decrease of saccadic amplitudes over viewing time (Pannasch et al., 2008; Unema et al., 2005). This pattern has been interpreted as a shift from an ambient to a focal processing mode, where ambient processing happens early and extracts global aspects of a scene and focal processing occurs later and extracts visual detail. Others have shown that low-level features with high spatial frequencies better predict the targets of saccades with shorter amplitudes, which likely map onto the focal mode (Tatler et al., 2006). More recent findings in macaques have revealed a decrease in fixations switching between different objects and an increase in successive fixations within the same object across viewing time (Ito et al., 2017).

Another line of research has shown a high incidence of refixations towards previously attended regions in complex scenes (e.g., 25% in natural scene recognition; Mannan et al., 1997; 35% in scene memorisation; Zelinsky et al., 2011). These findings generally deviate from the well-established hypothesis of Inhibition-of-Return (IOR), which describes a general difficulty of orienting towards previously attended areas (Posner & Cohen, 1984). While there is good evidence for IOR in search tasks with sparse visual information (e.g., Gilchrist & Harvey, 2000; McCarley et al., 2003), accumulating evidence suggests that it might not apply in the same way to gaze behaviour towards more visually rich scenes. For example, Henderson and Smith (2009) found the probability to saccade to preceding fixation locations was greater than or the same as other equally distant areas when memorising complex scenes. The authors referred to this as Facilitation-of-Return. Contemporary models of visual attention with an IOR component (e.g., Itti & Koch, 2000; Navalpakkam & Itti, 2005) may compromise model performance by overly penalising return saccades (Kümmerer & Bethge, 2021). One function of refixations may be that they facilitate rehearsing the memory of previously fixated locations or account for information that has previously been missed or processed insufficiently. Refixations lead to better accuracy in a recognition test after freely viewing scenes containing multiple objects (Zelinsky et al., 2011) and participants are much more likely to use refixations rather than working memory under most conditions (Draschkow et al., 2021; Droll & Hayhoe, 2007). Recent work has shown that refixations are frequently emerging in humans and non-human primates across different visual search paradigms and naturalistic task-related behaviour (Zhang et al., 2022).

Together, the above studies suggest that fixations may fall into different classes, according to their spatio-temporal characteristics in an object-based framework. We propose a simple algorithm that distinguishes three classes of fixations towards static and dynamic scenes: Detection fixations (D), which foveate a given visual object for the first time and thus allow the exploration of objects in a scene; Return fixations (R), which come back to a previously foveated object from elsewhere; Inspection fixations (I), which land on the same object as the previous fixation and likely target specific details within a given object.

Previous studies described shifts of fixation properties over viewing time (Pannasch et al., 2008) or with task demands (e.g., Droll & Hayhoe, 2007). Others studying eye movements during reading have identified fixation categories resembling those proposed here (first fixations, refixations and regressions, see above; Engbert et al., 2002; Vitu & McConkie, 2000). We propose to explicitly classify individual fixations in an object-based manner and according to their spatiotemporal aspects. One strength of this approach is that it is applicable to dynamic as well as static scenes (Mengers et al., 2025; Roth et al., 2022, 2023, 2024). Here we focus on its application in the context of free-viewing static natural scenes, a paradigm which has served as a testbed for most salience models (Harel et al., 2007; Itti & Koch, 2000; Kümmerer et al., 2016). In a first instance, this requires knowledge about the distribution of objects in each scene (which became image-computable in recent years; Ravi et al., 2024), but supplementary analyses show that—for a diverse set of natural scenes—the D, I, and R classification can also be approximated with a simple spatial heuristic.

The D, I, R classification allows the differential study of either type of fixation, e.g., regarding the weight of low-level, geometric and different semantic image features attracting them (Xu et al., 2014), or the effects of task or trial duration.

### Aim of the Present Study

First, we define criteria for classifying object-directed fixations as either D, I or R. We then test systematic differences between these classes by probing the consistency of differences between D, I, and R fixation maps across observers. Further, we test whether D, I, and R have differential profiles of semantic salience. Specifically, we compute the proportion of fixated objects with a given semantic feature and explore the weight of these features for predicting fixations (Xu et al., 2014), separately for D, I, and R. Third, to replicate and expand work on (focal and ambient) viewing modes, we test how D, I, and R fixations vary over trial duration and the effect of varying trial durations. We hypothesize that restricting viewing time leads to a prioritization of exploration over exploitation and thus an increase of Detections over Inspections and Returns. Fourth, in a supplementary analysis, we evaluate D, I, and R fixation classifications derived from a simple heuristic based on Euclidean fixation distance, rather than explicit knowledge about object locations in a scene. Finally, we make a well-documented implementation of our algorithm available.

We evaluated the D, I, and R approach on fixations from 144 observers freely viewing hundreds of complex scenes containing thousands of objects. To foreshadow our findings, across observers, fixation maps are significantly more consistent within than across D, I, R fixations; the prevalence of fixated objects with different semantic features varies across D, I, and R, with significant differences in the feature weights predicting each type of fixation in an established salience model (Xu et al., 2014); the proportion of D, I, and R fixations shifts over time, in line with earlier findings on ambient and focal viewing modes; shorter trial durations lead to an increase in D over I fixations and surprisingly, also to an increase in R fixations; finally, D, I, and R fixation can be well approximated with a spatial heuristic agnostic to object information (see Supplementary Materials). Taken together, our findings show that the D, I, R classification captures systematic differences in object-directed gaze behaviour in an intuitive and computationally feasible manner, which can be applied to datasets of static and dynamic scene-viewing.

## METHODS

### Subjects

#### 3 s Free-Viewing Condition.

One-hundred-and-one participants were recruited at Leibniz Institute of Psychology Information (ZPID) via their PsychLab offline service (*M*_*age*_ = 25.17; *SD* = 5.50; 7 left-handed; 72 females).

#### 2 s Free-Viewing Condition.

Forty-three participants were recruited at Justus Liebig University Giessen (*M*_age_ = 23.37 years; *SD* = 4.19; three left-handed; 33 females) and received course credit or 7 €/h for participation.

All participants had normal or corrected-to-normal vision. The study was approved by the local ethics committee, adhered to the Declaration of Helsinki and all participants gave informed consent.

### Apparatus

#### 3 s Free-Viewing Condition.

Stimuli with a resolution of 1200 × 900 pixels (29.6 × 22.2 dva) were displayed on a BenQ XL2430T monitor with a resolution of 1920 × 1080 pixels (47.4 × 26.7 dva). People were sitting at a distance of 64 cm from the screen.

#### 2 s Free-Viewing Condition.

Stimuli were presented on an ultra-high-definition monitor (3840 × 2160 resolution; LG Corporation, Seoul, South Korea) with a resolution of 2400 × 1800 pixels and 34.3 × 25.7 dva. The viewing distance was 55 cm.

The experiment was created and implemented with MATLAB Version R2019a (MathWorks, Natick, MA) using the Psychtoolbox Version 3.0.12 (Kleiner et al., 2007; Pelli, 1997). Eye movements were recorded using a desk-mounted Eyelink 1000 Plus (SR Research, Ottawa, Canada) at 1 kHz.

### Stimuli and Procedure

We used the OSIE dataset (Xu et al., 2014), which comprises a total of 700 complex everyday scenes and corresponding pixel masks for 5551 objects with binary labelling for 12 semantic attributes. As in previous analyses (Linka & de Haas, 2020), to prevent excessive overlap between the labels we removed the *Face* label from all objects with the *Emotion* label; the *Smell* label from all objects with a *Taste* label; and the *Operable* and *Gazed* label from objects with *Touched* label and finally the *Watchable* label from all objects with the label *Text*. Please note that for the present analyses, we removed any overlap between pixel masks. In cases of occlusion, object masks were separated such that if one object occluded another, the pixels of the foreground object were only registered as that object and excluded from the pixel mask of the underlying, occluded object.

For details of the design, see Linka and de Haas (2020). Briefly, after completing a 9-point calibration, participants freely viewed 700 images in 7 blocks of 100 images each. Each image was presented at the centre of the screen for 3 seconds (3 s free-viewing condition) or 2 seconds (2 s free-viewing condition) with a self-paced fixation disk in between trials. In a given condition, all images were presented in the same order across participants.

### Analysis

#### Data Processing.

All statistical analyses were performed in MATLAB R2019a (MathWorks). To exclude onset fixations (initiated before image onset), we excluded all fixations with an onset time below 100 ms. Further, we excluded fixations with a duration below 100 ms, following recommendations by the manufacturer. We used the pixel masks provided with the OSIE dataset to label fixations according to the objects they coincided with and the semantic labels of these objects. To prevent fixations from being classified according to our D-I-R schema based on overlapping object masks (e.g., masks layered on top of one another, such as text on a book cover), we separated each mask from the others. In case fixations fell on or within a distance of ∼0.5 dva from a labelled object, they were assigned the corresponding label.

#### Labelling Detection, Inspection and Return Fixations.

Our algorithm assigns D, I, and R labels for fixations towards objects the following way: A fixation is labelled *Detection* when it is the first fixation landing on a given object in the image; any fixation falling on the same object as the preceding one is labelled *Inspection*; any fixation towards a previously fixated object that follows after one or more intermittent fixations elsewhere is labelled *Return*. Please note that a single fixation can potentially be attributed to fall on multiple objects because of the included error margin (∼0.5 dva see above). Therefore, D, I, and R labels are not mutually exclusive and any single fixation may receive multiple labels. For an overview of the proportion of shared labels within each fixation type, see Supplementary Materials Figure S2A. Moreover, to examine whether our findings are sensitive to the use of the tolerance margin, we repeated all analyses using fixation labels assigned without any tolerance. This approach removed overlap between Detection, Inspection, and Return labels. Overall, the results closely matched those from the analyses using a 0.5 dva error margin (for examples, refer to Supplementary Materials Figures S3 and S4). Notable differences emerged only in analyses examining the temporal evolution of D, I, and R proportions over the course of free viewing, as well as in the overall distribution of fixation types. These differences likely reflected the mislabelling of (Inspection) fixations close to the edges of an object as background fixations when applying no error margin. They are detailed below and in Supplementary Materials Figure S5. Finally, for descriptive purposes, we plotted the distribution of object-mask sizes and object counts across images, along with the corresponding proportions of D, I, and R fixations (see Supplementary Materials Figure S2B–D).

#### Code Availability.

We provide MATLAB code and a manual for classifying fixations based on the D, I, R scheme and for generating corresponding fixation maps using the object-based or agnostic approach (see Supplementary Materials; osf.io/frvwd). This implementation requires only basic input data, such as the X and Y coordinates of fixations per image (as well as fixation durations and onset times for optional analyses of fixation dynamics).

#### Consistent Differences Between D, I, and R Fixations Across Observers.

**Figure 1.**
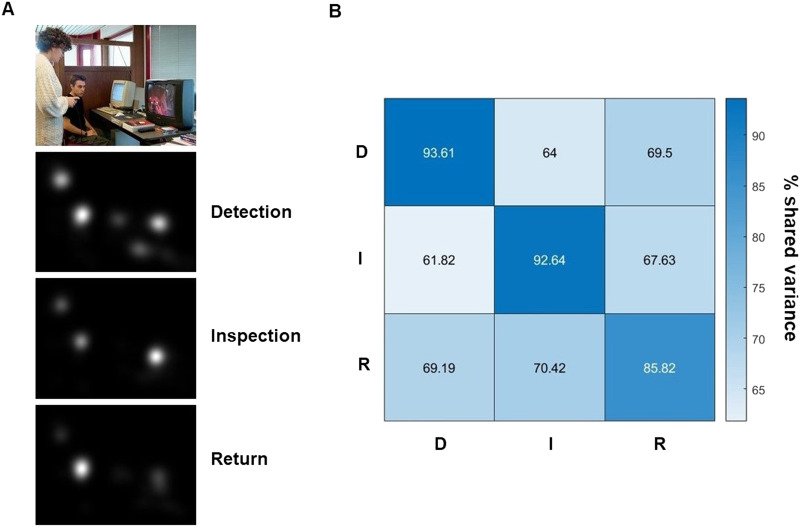
Shared variance between D, I, and R fixation maps. (A) Fixation maps of D, I, and R fixations for an example image. (B) Similarity matrix showing the mean shared variance between D, I, and R fixation maps across observers.

To test the consistency of differences between D, I, and R fixations across observers, we generated fixation maps for each fixation type and image, separately for odd- and even-numbered observers from the 3 s dataset. We then computed Pearson correlations between these fixation maps across the two groups of observers, both within and across D, I, and R maps. The resulting correlation matrices were averaged across images (Fisher *z*-transformed before averaging and then back-transformed) and squared in order to retrieve the shared variance (*R*^2^) of fixation locations within and between fixation classes. To test the consistency hypothesis, we used a bootstrapping approach to compare *z*-transformed correlations of fixation maps within a given class *versus* those between classes (i.e., the diagonal and off-diagonal entries in correlation matrices; Figure 1B below). Our aim was to test the null hypothesis that there are no significant differences between the diagonal and off-diagonal entries of the correlation matrix. In each iteration of the bootstrap analysis, we randomly selected a subset of 2,100 correlation coefficients from the whole correlation matrix and computed their mean. This process was repeated 10,000 times, resulting in a null distribution, against which we compared the observed mean correlation for the diagonal entries to. Specifically, we calculated the *p*-value by determining the proportion of randomly generated subsamples that were more extreme than the observed diagonal mean. This approach maximizes statistical power and controls the risk of Type I errors when testing groups with unequal sample sizes.

#### Diverging Feature Weights for D, I, and R Salience.

We also computed the prevalence of different object features among D, I, and R fixations and compared them between classes. Specifically, we computed the dwell time proportion of fixations landing on objects with the semantic feature labels *Face*, *Emotion*, *Touched*, *Gazed*, *Motion*, *Taste*, *Text*, and *Watchable*, separately for D, I, and R fixations. We then computed a 3 × 8 RM ANOVA with Fixation type (D, I, and R) and Semantic feature (*Face*, *Emotion*, *Touched*, *Gazed*, *Motion*, *Taste*, *Text*, and *Watchable*) as within-subject factors to test the Fixation type × Semantic feature interaction and follow-up post-hoc comparisons. We chose these features based on their strong saliency as reported by Xu et al. (2014). Please note that we do not report or interpret a main effect of Fixation type. Such an effect would not capture differences in overall D, I, and R, since (1) semantic features are normalized within each fixation type, and (2) individual fixations can be labelled with multiple types, introducing overlap that would bias the main effect. To account for individual differences in semantic salience (de Haas et al., 2019), we additionally ran a Linear Mixed Model including by-subject random slopes for dwell time proportions across D, I, and R for all semantic object categories (see Supplementary Materials for details). To further compare salience profiles between D, I, and R, we trained and tested the saliency model by Xu et al. (2014) separately for maps of D, I, and R fixations. This model is based on a linear SVM, which learns the weights of low-level pixel-wise features, as well as geometric and semantic features of objects to predict fixations (for details, see Xu et al., 2014). The weights were normalised to a common scale (0–1) for each fixation type so they reflect the relative weight within each type. Moreover, we compared the aggregated model weights for low-, mid-, and high-level features. For this analysis, we first computed combined salience maps for low-level features (i.e., *Colour*, *Intensity*, and *Orientation*), mid-level features (i.e., *Size*, *Complexity*, *Convexity*, *Solidity*, and *Eccentricity*) and high-level features (*Face*, *Emotion*, *Touched*, *Gazed*, *Motion*, *Sound*, *Smell*, *Taste*, *Touch*, *Text*, *Watchable*, and *Operability*), based on previously learned model weights. Then, we computed a second pass, learning the weights for the respective combined feature maps (low-, mid- and high-level), separately for each fixation type and for a control condition collapsing all fixations (cf. Xu et al., 2014). Finally, to evaluate the performance for models trained on D, I, R and for the control model collapsing all fixations, we computed their receiver operating characteristic (ROC) for predicting the respective fixation maps. For the ROC analysis, the true positive rate was plotted as a function of the proportion of pixels labelled as salient in the salience maps (i.e., as a function of the criterion).

#### Shifts Between D, I, and R Fixations Across Viewing Time.

To test their dynamics, we also analyzed the dwell time proportion of D, I, and R fixations as well as fixations that were not given any label (background fixations) as a function of viewing time for a given image. For this analysis, we first computed the dwell time proportion of labelled fixations for D, I, R and unlabelled fixations from 150 to 3000 ms viewing time using adjacent, non-overlapping 50 ms bins. Moreover, we examined the fixation duration and Euclidean distance to the previous fixation across viewing time, separately for D, I, and R fixations. For this analysis, we again computed the respective measure using adjacent, non-overlapping 50 ms bins from 150 to 3,000 ms. Please note that for all analyses probing shifts across viewing time we included all fixations overlapping with a given time bin. We further compared the overall dwell time proportion between D, I, and R for the full trial duration, using paired *t*-tests. The corresponding *p*-values were adjusted using the Bonferroni correction.

#### Probing Differences in D, I, and R Between Trial Durations.

Finally, we tested whether shortening trials has an impact on the dwell time proportion of D, I, and R fixations. Participants freely viewed the same 700 images, but this time for only 2 instead of 3 seconds each. We analysed the data by calculating the dwell time proportion of D, I, and R fixations from 150 to 2,000 ms using adjacent bins of 50 ms for participants in both the 3-second and 2-second conditions. Then we compared the dwell time proportions of D, I, and R fixations for each individual and condition using a bootstrapping approach; for each iteration, we combined fixation data from all 144 observers and randomly selected a subset of size *n* = 43 from the combined data. We then computed the statistic of interest based on the selected fixation data. We repeated this process 10,000 times, resulting in a sampling distribution of 10,000 bootstrap estimates. Assuming the null hypothesis (that there are no systematic differences between the 3 s condition and the 2 s condition in either D, I, and R proportions), a given dwell time proportion for the 2 s free-viewing condition should not be extreme compared to this bootstrapped null distribution. To calculate the *p*-value for the observed statistic of interest, we determined the proportion of randomly generated subsamples from the combined groups that were more extreme than the observed value. This method maximizes statistical power and controls the risk of Type I errors for unequal group sizes. All *p*-values were adjusted using the Bonferroni correction.

## RESULTS

First, we tested consistency within and differences between D, I, and R fixations across observers. Figure 1B shows the mean shared variance between fixation maps of each class. D, I, and R fixation maps showed a high degree of consistency across observers (94%, 93%, 86% shared variance across observers, respectively).

The shared variance *between* D, I, and R fixation maps was 67% on average. This difference was highly significant (bootstrapping test confirming more similar fixation maps within than between fixation classes, *p* < .001).

### Differential Salience for D, I, and R Fixations

**Figure 2.**
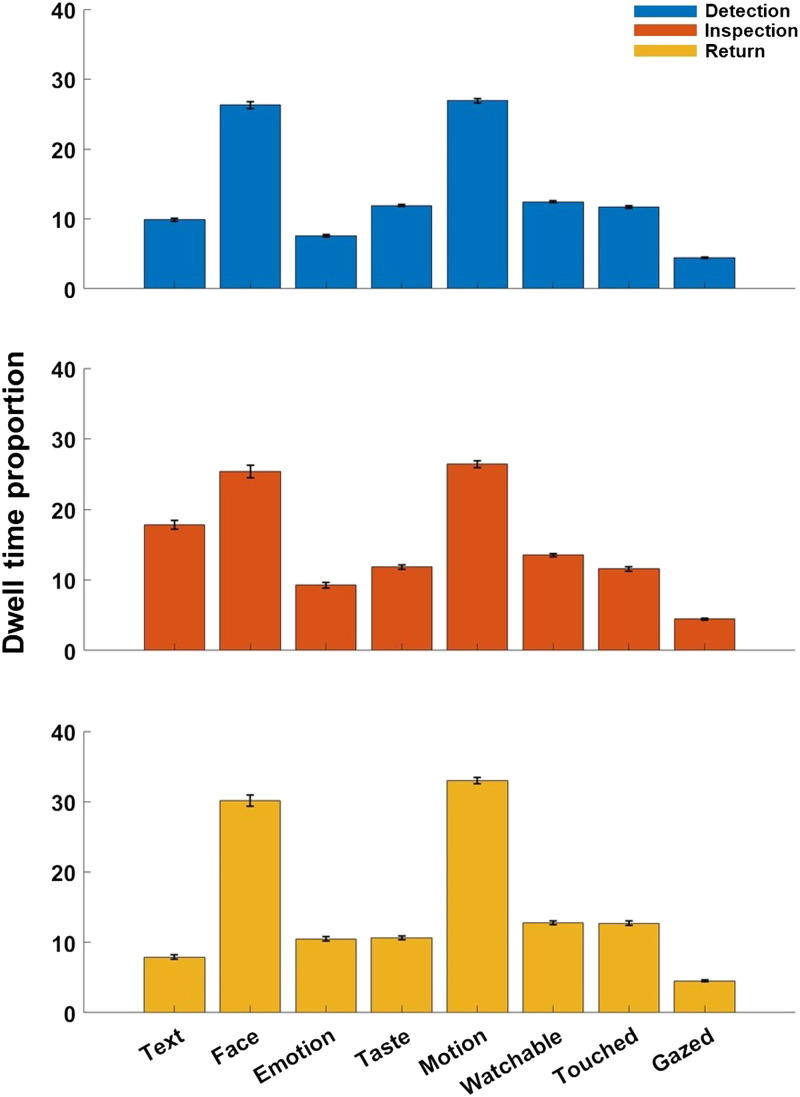
Proportion of D, I, and R for fixations landing on different semantic features. Bar plots displaying the proportion of cumulative dwell time on objects of a given semantic feature for D, I, and R respectively. Error bars indicate the 95% CI across observers. Fixation types are indicated by colour as shown in the inset.

To examine potential differences in D, I, and R salience, we examined the relative prevalence of objects with a given semantic feature, separately for each fixation class. For this, we computed the proportion of cumulative dwell time for objects of each semantic feature, separately for D, I, and R (Figure 2). The proportions of D, I, and R fixations varied substantially across labels. For instance, fixations landing on *Text* were primarily Inspections (18%; with 10% Detection and 8% Returns), but fixations towards faces with *Emotion* were primarily Returns (10%) and Inspections (9%; with 8% Detections). A 3 (Fixation Type; D, I, and R) × 8 (Feature dimension; *Face*, *Emotion*, *Touched*, *Gazed*, *Motion*, *Taste*, *Text*, and *Watchable*) repeated-measures ANOVA for cumulative dwell time proportion showed a significant Fixation type × Feature interaction, *F*(14, 1400) = 359.47, *p* < .001. Follow-up pairwise comparisons between D, I, and R for each semantic label using the Tukey-Kramer test showed significant differences between D, I, and R fixations for all semantic dimensions (*p* < .05), but *Gazed* (D vs. I: *p* = .74; D vs. R: *p* = .23; I vs. R: *p* = .55), *Taste* between D and I (*p* = .91), *Motion* between D and I (*p* = .06) and *Touched* between D and I (*p* = .41). Using a Linear-Mixed Model approach that accounted for individual differences in dwell time across D, I, and R, as well as salience for object semantics (de Haas et al., 2019), confirmed the pattern of findings from the analyses above (see Supplementary Methods and Results).

**Figure 3.**
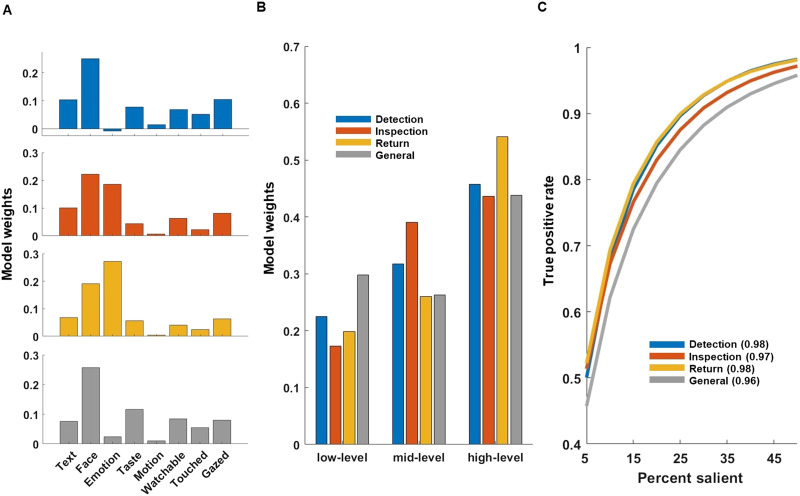
Model performance and learned weights for eight semantic dimensions. (A) Bar plots showing the normalised model weights for eight semantic dimensions retrieved from four models trained on D, I, R and all fixations collapsed. (B) Bar plot showing the normalised model weights for low-, mid-, and high-level features respectively for D, I, R and all fixations collapsed. (C) ROC curves of models trained with D, I, R or all fixations. AUC values are presented in parentheses. Fixation types are indicated by colour as shown in the inset.

In a next step, we used a linear SVM as part of an established salience model by Xu et al. (2014), to retrieve the weights for pixel-, object-, and semantic-level attributes for predicting D, I, and R fixations separately, as well as for predicting all fixations in a collapsed (i.e., traditional) manner. Figure 3A shows the weights for eight semantic dimensions across D, I, R fixations as well as for all fixations collapsed.

Consistent with the data by Xu et al. (2014) using fixation maps including all fixations, the dimensions *Face* and *Text* carried the highest weights, followed by *Gazed*, *Watchable*, and *Taste*. Across D, I, and R fixations, we found that the weights could vary substantially. For example, the weight of the dimension *Emotion* was an order of magnitude higher for Returns (0.27) and Inspections (0.19) than for Detections (−0.01), suggesting that the expressiveness of a face determines the likelihood of a face being inspected further and revisited, but not of it being fixated in the first place. The weight of *Watchable* was higher for Detections (0.07) and Inspections (0.06) than for Returns (0.04), and a similar pattern was observed for the weight of *Text* (Inspections 0.1, Detections 0.1, and Returns 0.07). This indicates that displays and text are salient items attracting close inspection and typical reading behaviour, with multiple successive fixations and small saccades, but are less likely to be revisited thereafter. Generally speaking, these differences indicate that distinct fixation types are guided by different semantic object information—patterns that are obscured when all fixations are collapsed into a general model. To compare the weights between low-, mid- and high-level features in predicting D, I, R and all fixations collapsed, we performed a second training based on the combined salience maps for each level and fixation type. Figure 3B shows the weights for combined low-, mid-, and high-level features for each fixation type as well as for all fixations collapsed (general model). Across all models, weights were generally lowest for low-level features, followed by mid-level and high-level features. However, the distribution of feature-level weights varied considerably between D, I, and R. Interestingly, for Inspections, the weight of mid-level features was substantially larger than for the other fixation classes, while that of low- and high-level features was somewhat lower. For Returns, the weight of high-level features was substantially higher than for Detection and Inspection fixations. This suggests that high-level semantic information is the strongest determinant of returning to this object later on. At the same time, the likelihood of multiple successive fixations on a given object more strongly depends on mid-level, geometric features. A follow-up exploratory analysis showed that Inspection frequency was correlated with object size (*r* = .36, *p* < .001), reminiscent of the more frequent refixations observed in longer words during reading (Engbert et al., 2002; Vitu & McConkie, 2000).

To test the accuracy of model predictions based on our approach with a traditional metric, we computed cross-validated receiver operating characteristic (ROC) curves (see Xu et al., 2014 for details), separately for the D, I, and R predictions as well as for the prediction of collapsed fixations. Figure 3C shows the ROC curves for models predicting D, I, and R fixations as well as for that predicting all fixations. The area under the curve for specific predictions of Inspection fixations (0.97) was close to that for Detections (0.98) and Returns (0.98). Importantly, all AUC scores for D, I, R predictions were higher than those for the general model (0.96), despite relying on sparser data and aiming for more specific predictions. This underscores that the specific predictions these models aim for indeed target meaningfully distinct categories of fixations with specific salience profiles, which are obscured when conflating these different types of fixations.

### D, I, and R Fixations Across Viewing Time

**Figure 4.**
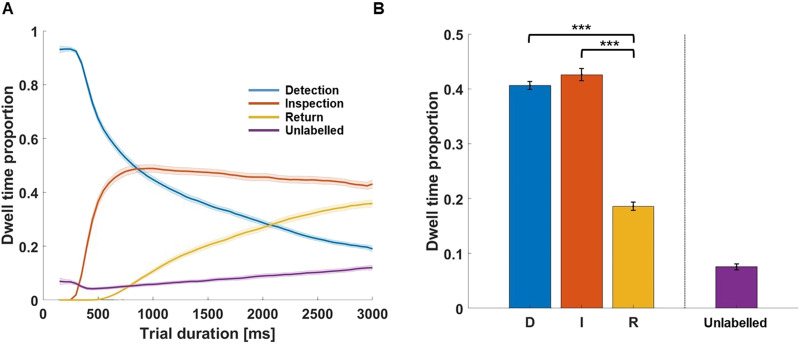
Proportions of fixations across trial duration. Line plots depicting mean fixation proportions (A; *y* axis) across observers for D, I, R and unlabelled fixations observed for the full trial duration starting from 150 ms to 3000 ms (*x* axis). The proportions were calculated using adjacent, non-overlapping 50 ms bins. Shades and error bars indicate the 95% CI across observers. Panel B shows the proportion of D, I, R and unlabelled fixations across all subjects and for the full three seconds viewing time. Fixation types are indicated by colour as shown in the inset. Unlabelled fixations fell on image regions with no labelled object. *p < .05, **p < .01, and ***p < .001.

Further, we analysed the temporal patterns of D, I, and R fixations across the trial duration (3 s). For this, we computed the dwell time proportion for D, I, R and unlabelled fixations using adjacent, non-overlapping 50 ms bins, with unlabelled fixations pertaining to fixations falling on image regions without a labelled object. We expected a descending dwell time proportion of Detection fixations and an ascending proportion of Inspections and Returns across viewing time, in line with the hypothesis of ambient and focal modes. Figure 4A shows the dwell time proportion of fixations for D, I, R and unlabelled fixations across viewing time.

As expected, Detection fixations occurred most frequently during the first 850 ms, peaking at 250 ms before slowly decreasing. The dwell time proportion of Inspections raised steeply, before plateauing at around 800 ms. Returns showed a quasi-linear increase from 500 ms after stimulus onset. Finally, the dwell time proportion of unlabelled fixations showed a slow quasi-linear increase from 400 ms after stimulus onset.

We further compared the overall dwell time proportion of D, I, R and unlabelled fixations for the full 3 s viewing time (Figure 4B). No significant difference was found between D and I fixations, *t*(100) = −2.4, *p* = .06. Further observers showed larger overall dwell time proportions for D than R, *t*(100) = 38.18, *p* < .001 and for I than R fixations, *t*(100) = 28.44, *p* < .001. The dwell time proportion of unlabelled fixations was 0.08 and hence considerably smaller than those for D, I, and R.

Further analyses, in which object labels were assigned to fixations without applying a tolerance margin—and thus avoiding the assignment of multiple D, I, and R labels to a single fixation—showed a slightly different pattern of results. The proportion of Inspection fixations still rose strongly early in the trial, plateauing around 900 ms, but at a noticeably lower level (∼0.35 vs. ∼0.45) compared to the version with a margin. Unlabelled fixations remained low early on, but increased steadily, reaching a higher level by the end of the trial. This shift is also reflected in the overall dwell time proportions (Supplementary Materials Figure S5D), with fewer Inspection fixations and a corresponding rise in unlabelled fixations when no margin was applied. This shift from inspection to unlabelled fixations likely reflects minor eye-tracking inaccuracies and the consequential mislabelling of Inspection fixations close to the edges of an object as background fixations when applying no error margin (see Supplementary Materials Figure S5).

**Figure 5.**
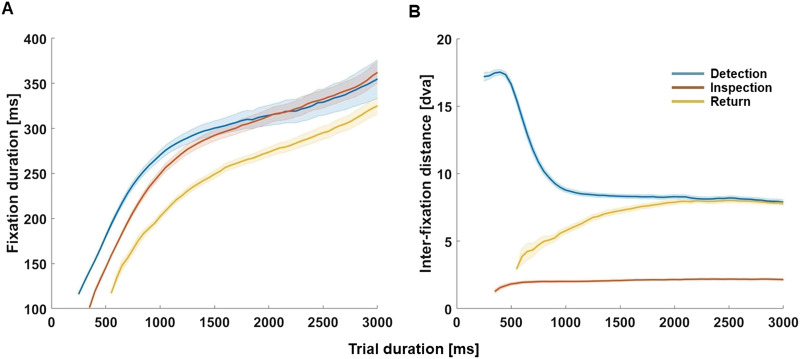
Fixation duration and Euclidean distance to previous fixation across trial duration for D, I, and R. Line plots depicting mean fixation duration (A; *y* axis) across observers for D, I, and R fixations observed for the full trial duration starting from 150 ms to 3000 ms (*x* axis). Fixation duration was calculated using adjacent, non-overlapping 50 ms bins. Panel B shows a line plot depicting the mean Euclidean distance to the previous fixation for D, I, and and R over viewing time (bins of 50 ms). Shades indicate the 95% CI across observers. Fixation types are indicated by colour as shown in the inset.

Furthermore, we examined fixation duration and Euclidean distance to the preceding fixation over trial duration. Again, we averaged fixation duration and preceding fixation for non-overlapping and adjacent time bins of 50 ms. Based on work showing an increase in fixation duration and a decrease of saccadic amplitude during free viewing natural scenes (Unema et al., 2005), we expected the mean fixation duration to increase for D, I, and R and the Euclidean distance to the previous fixation to decrease over time for D and R. Figure 5 shows the mean fixation duration (A) and mean Euclidean distance to previous fixation (B) across trial duration.

As expected, the fixation duration increased across trial duration for D, I, and R, most steeply during the first 1050 (D) to 1600 ms (R). The mean Euclidean distance to previous fixation decreased for Detection before plateauing around 1000 ms. Interestingly, the corresponding distance *increased* for Returns to reach a similar plateau around 2100 ms. Unsurprisingly, Inspection fixations had a generally much lower distance to preceding fixations, which only marginally increased over time.

### The Effect of Trial Duration on the Proportion of D, I, and R

**Figure 6.**
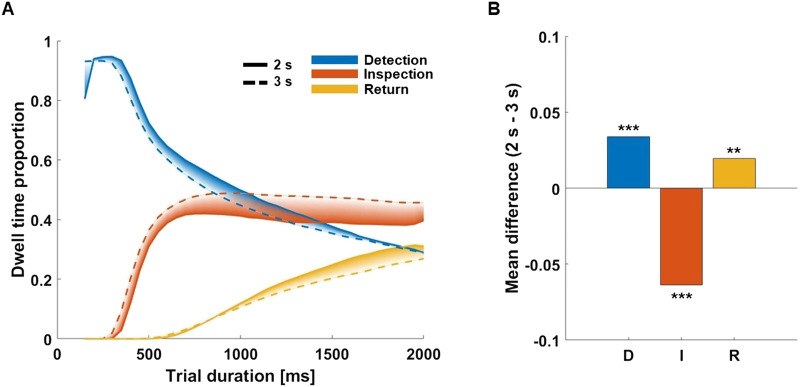
Differences in D, I, and R between 2 s and 3 s trial duration. Panel A shows line plots depicting mean fixation proportions (A; *y* axis) across observers for D, I, and R fixations observed for the 2 s condition (solid lines) and 3 s condition (dotted lines) starting from 150 ms to 2000 ms (*x* axis). The proportions were calculated using adjacent, non-overlapping 50 ms bins. Shades indicate the area between curves for a given label. Panel B shows the mean differences in D, I, and R proportion between the 2 s condition and the 3 s condition. Error bars show the standard error of a given class. Asterisks represent the *p*-values as retrieved from each bootstrapping test probing group differences between both conditions. Fixation types are indicated by colour as shown in the inset. *p < .05, **p < .01, and ***p < .001.

To test the effect of shorter trial durations (2 versus 3 s) on the proportion of D, I, and R fixations, we acquired additional data with a shorter viewing duration. We hypothesized that limiting the presentation time would lead to a more explorative viewing style with a higher proportion of D and lower proportions of I and R fixations. Figure 6A shows the proportion of D, I, and R across trial duration for the 3 and 2 s free-viewing condition. Figure 6B shows the overall mean differences in D, I, and R for both conditions.

Across trial duration D fixations showed a larger peak at around 300 ms and a less steep descent in the 2 s trial condition compared to the 3 s condition. Inspection fixations showed a lower peak at 800 ms in the 2 second vs. 3 second condition and stayed at a lower proportion across the remaining trial time. Surprisingly, the linear increase of Return fixations was similar in both conditions until around 950 ms, when—counter to our expectation—the slope became steeper in the 2 s condition.

Comparing the overall proportion of D, I, and R between free viewing conditions, we found that there was a larger proportion of D fixations (*M*_*diff*_ = 0.03, *p* < .001) and a lower proportion of I fixations (*M*_*diff*_ = −0.06, *p* < .001) in the 2 second condition vs. 3 second condition. Surprisingly, we also found a larger proportion of R fixations in the 2 s vs. 3 s condition (*M*_*diff*_ = 0.02, *p* < .01). Taken together, shorter viewing time led to an increase in the proportion of Detection and Return fixations at the expense of Inspection fixations. The unexpected increase in Return fixations was counter to our expectations of a simple shift towards more exploratory gaze.

## DISCUSSION

Traditional models of visual attention either focus on the spatial distribution of fixations across an image (e.g., heatmaps) or on their precise sequence in scan-paths. However, neither approach utilizes the object-directed nature of typical fixation sequences, nor allows for intuitive distinctions between different types of fixations. Here, we present a simple algorithm to sort fixations towards naturalistic scenes into three distinct classes. An evaluation on a large scene viewing dataset confirmed systematic and reliable differences between Detections, Inspections and Returns, including distinct salience profiles and dynamics.

We computed separate D, I, and R fixation maps for 700 naturalistic scenes using data from >100 observers and found reliable differences between these classes, which generalised across images and observers. Previous work has shown a shift in fixation duration and saccadic amplitude over free viewing time (ambient vs. focal mode; Pannasch et al., 2008; Unema et al., 2005) and examined the proportion of refixations under different task instructions and visuo-sensorimotor tasks (e.g., Droll & Hayhoe, 2007). The algorithm proposed here classifies each object-directed fixation according to its temporo-spatial placement within a sequence. This allows for addressing specific (functional) hypotheses regarding D, I, and R and provides an easy-to-generate metric to evaluate process-specific predictions and questions of practical interest. For instance, researchers and practitioners may be interested in modelling the specific likelihood of a visual object being fixated at all (say, a warning or exit sign), revisited multiple times (say, an advertisement) or a given object being inspected in more detail (say, an infographic).

Furthermore, the proposed D, I, R approach opens the possibility to capture the dynamic aspects of object-directed gaze towards dynamic stimuli, and has already been used in this context successfully (Mengers et al., 2025; Roth et al., 2022, 2023, 2024). Notably, the D, I, R approach overcomes the problem that traditional, string-based methods for scan-path comparisons (Cristino et al., 2010) become computationally infeasible when visual content is moving and thus dissociating from pixel space, which necessitates frame-wise temporal resolution (Roth et al., 2023).

Previous work has demonstrated that scene fixations can be predicted by a set of 8 semantic object dimensions (i.e., *Faces*, *Emotions*, *Touched*, *Gazed*, *Motion*, *Taste*, *Text*, and *Watchable*), outperforming low-level and other object-level attributes (Xu et al., 2014). We explored differences in the proportions of fixations attracted by these features and found that they varied significantly across D, I, and R fixations. This was also reflected in the weights for each semantic attribute in predicting D, I, and R salience fitting the model by Xu et al. (2014). For example, the proportion and weight of the feature *Text* was high for Inspections, which is most likely due to successively foveating word segments while reading. Whereas faces carried a very high weight, in particular for Detection fixations, *emotional* expressions only did so for Inspections and Returns. Extrafoveal faces in scenes appear to strongly attract saccades, regardless of their expression. But once fixated, the expressiveness of a face strongly modulated the tendency for successive Inspection and Return fixations. This may also reflect a tendency to sample distinct facial regions when looking at emotional (vs. neutral) expressions (Schurgin et al., 2014). These findings suggest that D, I, and R fixations may serve distinct perceptual functions during natural viewing, depending on the semantic nature of the target object. Future research should test this hypothesis by systematically manipulating object semantics and measuring how D, I, and R proportions vary. This could involve controlled experiments where object meaning, relevance, or contextual associations as well as low- and mid-level features are altered independently within the same stimuli.

Furthermore, we found that specific predictions of D, I, and R fixations outperform the performance of a generic model including all fixations. Inspection fixations showed a higher weight for mid-level geometric features than other fixations while Detection fixations showed a higher weight for high-level features than other fixations. The finding that high-level features carry a particularly high weight for Detection fixations extends earlier results on the time-course of low- vs. high-level salience. The influence of low-level salience quickly declines with the latency of the immediate saccade (Anderson et al., 2015) and over the course of a trial (Xu et al., 2014). The D, I, R algorithm allows to separate the exploration of objects from Inspections and Returns independently of trial time. The current data show that this exploration is predominantly driven by high-level features, while Inspection fixations—successive fixations on the same object—are more dependent on geometric mid-level features. This suggests that semantic features are particularly important for the salience of extrafoveal targets, whereas subsequent fixations on a currently foveated object more strongly depend on geometric properties. Future research should use the D, I, R framework to explore such differences in feature weights in more depth to further test functional implications of D, I, and R fixations.

Past research suggests a shift from a global to a focal processing mode across viewing time, indicated by a decrease in saccadic amplitudes and increase of fixation durations (Ito et al., 2017; Pannasch et al., 2008; Unema et al., 2005). Our results corroborate and extend these findings by showing an increase in fixation duration across D, I, and R as well as a descending frequency of Detection fixations and an ascending but quickly plateauing frequency of Inspection fixations. The switch from Detection to Inspection on average happens at ∼1 second which corresponds to recent findings in macaque monkeys (i.e., switch from background to object and object to object saccades to intra-object saccades at ∼1 second of free-viewing (trial duration 5 s); Ito et al., 2017). Moreover, we found a quasi-linear increase of Returns across viewing time. Finally, we found a decrease in Euclidean distance to previous fixation for Detections, but not for Returns, showing that a decrease of saccadic amplitudes over free viewing time as e.g., reported by Pannasch et al. (2008) is driven by Detection fixations and does not hold for Return fixations. Return fixations on the contrary seem to be restricted to close-by objects early in a trial and the corresponding saccades triple their amplitudes over the course of the first two seconds. Future research should use the D, I, R scheme to determine in how far this change is due to dynamic changes in (relative) Return saliency of peripheral objects, *versus* an effect of possible Return locations building up with exploration.

Finally, we found shorter trial durations increase the proportion of Detections and decreases that of Inspections. This finding is in line with a hypothesized faster shift from exploitation to exploration when the overall viewing time is restricted. Surprisingly, we also found an increase in Return fixations when the duration of the trial is reduced. This suggests a simple exploit/explore dichotomy may be missing functionally meaningful differences captured by the D, I, R scheme’s distinction between Inspection and Return fixations. When pressed for time, observers prioritize Returns over Inspections, suggesting that the former is less dispensable than the latter. A potential explanation for this is that the interplay between Detection and Return fixations contextualizes objects within a scene. In shorter trials, the prioritization of understanding contextual object (inter-)relations may take precedence over Inspections as it can play an important role in more efficient scene comprehension (Murlidaran & Eckstein, 2025). Future experiments can test this hypothesis directly, e.g., by testing changes in D, I, R proportions as a function of object-scene congruency and viewing time (cf. Võ & Henderson, 2009). An alternative explanation could be that the increased pace of exploration, with Detections spread across more objects and fewer Inspections, may lead to incomplete processing and therefore require more Returns, akin to regressions in reading (Engbert et al., 2002; Vitu & McConkie, 2000).

By providing an easily accessible and ready-to-use algorithm for the D-I-R classification we hope this classification approach will be adopted and prove useful for researchers from diverse fields, such as cognitive science, neuroscience, basic and applied vision research. For instance, future research could investigate whether Detection, Inspection and Return fixations and saccades rely on partially distinct neural processes. Some have suggested separate neural streams to be associated with distinct visual processing modes; the dorsal stream favouring a global processing of the visual space appropriate for exploration, and the ventral stream, responsible for a more central inspection of objects appropriate for exploitation (Sheth & Young, 2016; see also Unema et al., 2005).

To identify D, I, and R fixations, we used pixel masks of objects in the images. Object segmentations have become image computable (Ravi et al., 2024), but still may not be available in all cases. Therefore, we tested whether D, I, and R fixations could be approximated with a simple spatial heuristic, which treated fixations with a Euclidean distance <10% of the image width as landing on the same ‘object’. We found that these content-agnostic labels predominantly matched those derived using object masks (74% of Detections, 78% of Inspections, and 65% of Returns) and yielded a very similar divergence of heatmaps (see Supplementary Materials). Here, we used a diverse set of complex natural scenes and found that 10% of the image width provided a good heuristic for inter-object separation. Other types of images with considerably sparser or denser distribution of visual objects may require adjusted spatial heuristics, which could be determined e.g., by labelling part of the stimuli or combining the D, I, R approach with the detection of region of interests via hidden Markov models (Coutrot et al., 2018).

The present findings provide evidence for systematic and reliable differences between Detection, Inspection and Return fixations and point to potentially diverging functional roles. Future studies should test the functional significance of each type of fixation in targeted experiments, e.g., probing the effect of working memory load or individual capacity on the proportion of R fixations. Future research could also investigate how individual differences in gaze behaviour (e.g., Broda & de Haas, 2022; de Haas et al., 2019; Linka & de Haas, 2020) are reflected in D, I, and R fixations and how they vary across age (Helo et al., 2014; Krishna et al., 2017; Linka et al., 2023, 2025). Further, oculomotor metrics like saccadic velocity and acceleration may reveal further facets of D, I, and R fixations and the differences between them. Finally, the D, I, R schema introduced here shows an interesting parallel to the well-established fixation taxonomy in reading research. First fixations on a word may be compared to object Detection fixations, refixations of a word to Inspection fixations and regressions to previous words in a text to Return fixations (Engbert et al., 2002; Vitu & McConkie, 2000). While dominant models of eye movements during reading emphasize the role of lexical and linguistic factors to explain e.g., regressions (Engbert et al., 2005; Reichle et al., 2009), future research may probe whether there is mechanistic overlap between D, I, R patterns during scene viewing and corresponding viewing patterns during reading.

Taken together, we find that the D, I, and R classification algorithm captures systematic and reliable differences between subgroups of fixations with diverging salience and dynamic profiles, suggesting they may map onto distinct functional classes of fixations. The approach has already been successfully applied to dynamic stimuli and for static stimuli without annotations, the D, I, and R classification can be approximated in a content-agnostic manner with a simple spatial heuristic. We provide a well-documented implementation of these algorithms that enables researchers to functionally classify fixations from any scene viewing dataset in a straightforward way.

## ACKNOWLEDGMENTS

We would like to thank Xu et al. (2014) for making their stimuli, annotations and code freely available. Further, we thank Stefanie Mueller and the PsychLab team at ZPID for their help with data collection.

## FUNDING INFORMATION

This research was supported by European Research Council Starting Grant 852885 INDIVISUAL; BdH was further supported by Deutsche Forschungsgemeinschaft (DFG, German Research Foundation) Project Nos. 222641018-SFB/TRR 135 TP C9 as well as “The Adaptive Mind”, funded by the Excellence Program of the Hessian Ministry of Higher Education, Science, Research and Art.

## AUTHOR CONTRIBUTIONS

M.L.: Conceptualization; Formal analysis; Writing – original draft. B.d.H.: Conceptualization; Formal analysis; Writing – review & editing.

## DATA AVAILABILITY STATEMENT

The data used in this paper for the 3 s condition were originally published in Linka and de Haas (2020). All anonymized data and MATLAB code to reproduce the presented findings are freely available at osf.io/frvwd.

## Supplementary Material


