# Supplementary material for: Detection, Inspection, Return: An Object-Based Classification and Metric of Fixations in Complex Scenes
Source: Open Mind (Camb). 2026 Jan 15;10:47–65. doi: 10.1162/OPMI.a.319 (PMC13053019; doi:10.1162/OPMI.a.319)
Supplement: Supplementary file 1 [file opmi-10-47-s001.pdf]

Supplementary Materials

Supplementary Methods

Object-agnostic approximation of D, I and R fixations

For an object-agnostic labelling of D, I and R fixations, we considered any fixation to be on the same object/area when the Euclidean distance to another fixation was less than 1/10th of the image width. Accordingly, a fixation is labelled as *Detection* when it was the first fixation within the corresponding radius; as *Inspection* when the distance to the preceding fixation was below this threshold; and as *Return* otherwise (some previous fixation closer than the threshold, but not the preceding one). D, I and R fixation maps were created by convolving the corresponding fixations with a gaussian kernel with a size corresponding to 1/16 of the image width. Finally, we z-standardized each map to zero mean and unit variance.

To test the accuracy of approximating D, I and R fixations based on their spatial distance, we computed the proportion of approximated labels that matched the corresponding labels determined with the ground-truth object pixel mask information.

Additional descriptives

Due to the applied error margin of 0.5 dva when assigning a fixation to a given object, multiple D, I and R labels could be assigned to a given fixation (see also Methods in the main text). To explore the overlap of D, I and R labels as well as object frequency over images, object size and the relationship between object frequency and D, I and R proportion, we performed several additional descriptive analyses.

D, I and R label overlap

To quantify the overlap among D, I and R fixation, we computed the proportion of fixations within each category that also received one or both of the other labels.

Object count distribution

To determine the distribution of object counts over images, we calculated the number of annotated objects per image using object metadata from the stimulus annotations.

D, I and R dwell time proportion by object count

To investigate how fixation behaviour (D, I, R) varied with the number of objects depicted in the scenes, we examined the proportion of dwell time attributed to each fixation class as a function of object count per image. For each image and participant, we computed the proportion of total dwell time for each fixation type. These proportions were then averaged across participants to receive mean D, I, and R proportions per image. Images were grouped by object count, and for each group with sufficient sample size (≥10 images), the mean was calculated.

Object Size Distribution

Finally, we quantified the size of all annotated objects in units of square degrees of visual angle (dva²). Object masks were binarized and summed, and total pixel areas were converted to dva².

Fixation labeling without tolerance margin

For analyses in which fixations were labelled based on the semantic category of the object they landed on, and classified as D, I or R, we applied a tolerance margin of 0.5 dva to account for potential measurement error and for the extent of the foveal region. To assess whether our findings are sensitive to this tolerance margin, we repeated all analyses without applying any tolerance margin when assigning semantic labels or D, I, and R classifications to fixations.

Probing differential salience for D, I and R using Linear Mixed-Effects Models

To probe differences in semantic salience for D, I and R with an alternative method, we computed a linear mixed-effects model (LMM) of dwell time with Fixation type (Detection, Inspection, Return) and Semantic feature (*Face*, *Emotion*, *Touched*, *Gazed*, *Motion*, *Taste*, *Text*, *Watchable*) as fixed effects. We tested the interaction between Fixation type and Feature on dwell time proportion. We further included random intercepts and random slopes for Feature by subject to account for individual differences in semantic salience (de Haas et al., 2019). The model was specified as:

Dwell Time Proportion ~ Fixation type * Semantic feature + (Semantic feature | Subject)

The dependent variable, dwell time proportion, was calculated by aggregating fixation data across all images for each subject. For every combination of Subject, Semantic feature, and Fixation type, we computed the total dwell time spent on a given feature and divided it by the subject's total dwell time for that fixation type across all labelled objects. This resulted in proportion values ranging from 0 to 1. The resulting analysis matrix contained one row per Subject × Fixation type × Semantic feature combination, with columns for Subject, Dwell Time Proportion, Fixation type and Semantic feature.

Both Fixation type and Semantic feature were modelled as within-subject predictors. The model was fit using restricted maximum likelihood (REML) in MATLAB. Significance of fixed effects was assessed using marginal Type III F-tests with Satterthwaite approximation for degrees of freedom. The interaction was followed up with pairwise comparisons between fixation types within each feature using linear contrasts of the fixed effects.

Supplementary Results

Approximating D, I and R without object information

We tested the accuracy of image-agnostic D, I and R classifications based on a simple spatial heuristic, using a critical inter-fixation distance corresponding to $1/10^{th}$ of the image width (see data processing, methods). Figure S1A shows original D, I and R fixation maps based on object-wise pixel masks (left-hand side) and the corresponding approximations (right-hand side) for an example image. Figure S1B shows the distribution of ground truth labels for each group of approximated labels, including the proportion of ‘correct’ matches.

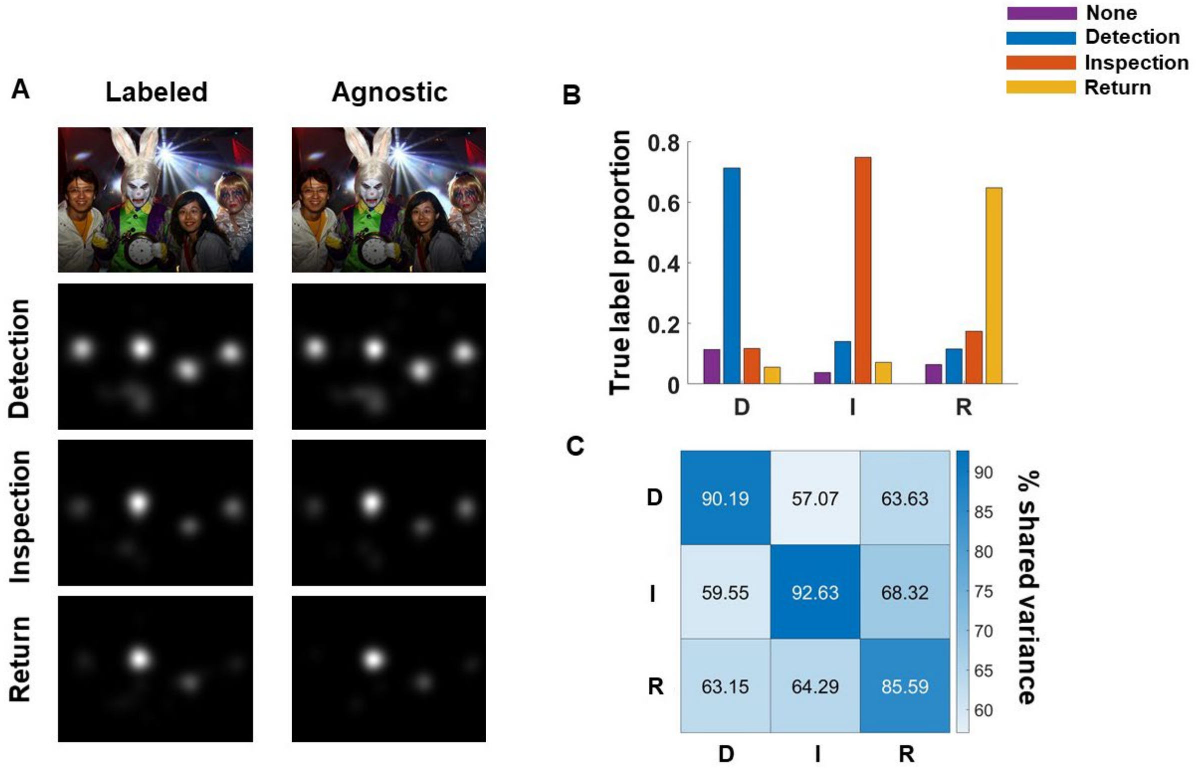

Figure S1. Object-agnostic approximation of D, I and R fixations. (A) fixation maps of an example image computed for D, I and R fixations determined based on object-wise pixel masks (left-hand side) and corresponding approximations based on spatial distance (right-hand side). (B) Histogram of ground-truth labels for approximated D, I and R fixations. Note that the ground truth analyses left background fixations unlabelled ('None', purple), while approximations necessarily labelled these as D, I or R. Colours indicate fixation types as shown in the inset. (C) Correlation matrix showing the mean shared variance between D, I and R fixation maps computed with object-agnostic approximations for one half of the observers vs. the other half.

We found that 71% of approximated Detections corresponded to actual Detections according to object metadata, 75% of Inspections and 65% of Returns. Probing the consistency within and differences between D, I and R fixation maps across observers indicated large consistencies for approximated D, I and R fixation maps, sharing 90% (Detections), 93% (Inspections) and 86% (Returns) of their variance on average, but only 63% between these maps. This difference was highly significant (bootstrapping test confirming more similar fixation maps within than between fixation types, $p < .001$; Figure S1C).

Additional descriptives

To explore the overlap of D, I, and R labels as well as object frequency over images, object size, and the relationship between object frequency and D, I, and R dwell time proportions, we performed several descriptive analyses.

D, I and R label overlap

The overlap of fixation classifications across D, I and R is shown in Figure S2A. Fixations labelled as Detections were uniquely assigned that label in the majority of cases (84%), while 12% were also classified as Inspections and 4% as Returns. Among Inspection fixations, 82% were exclusive to that category, 12% overlapped with Returns, and 6% with Detections. For Returns, 76% were uniquely labelled, with 10% overlapping with Detections and 14% with Inspections.

Object count distribution

The number of annotated objects across images is depicted in Figure S2B. The number of annotated objects per image ranged between 2 - 27 objects and followed a right-skewed distribution. Most images contained between 6 - 7 objects, with fewer images containing very high or very low numbers of objects. This indicates a relatively consistent object distribution across the image set, albeit with a long tail.

D, I and R dwell time proportion by object count

Figure S2C shows the relative proportion of dwell time for D, I and R fixations as a function of object count per image. Dwell time proportions for Detections increased with higher object counts, while proportions for Inspections showed a slight decreasing trend. Return fixations maintained relatively stable proportions across object counts. These trends suggest that increased object richness may shift fixation behaviour toward more exploratory (i.e., Detection-based) patterns, while diminishing Inspections.

Object size distribution

Figure S2D shows the positively skewed distribution of object sizes, with many small objects and a progressively lower frequency of larger objects (*Mdn* = 11.76).

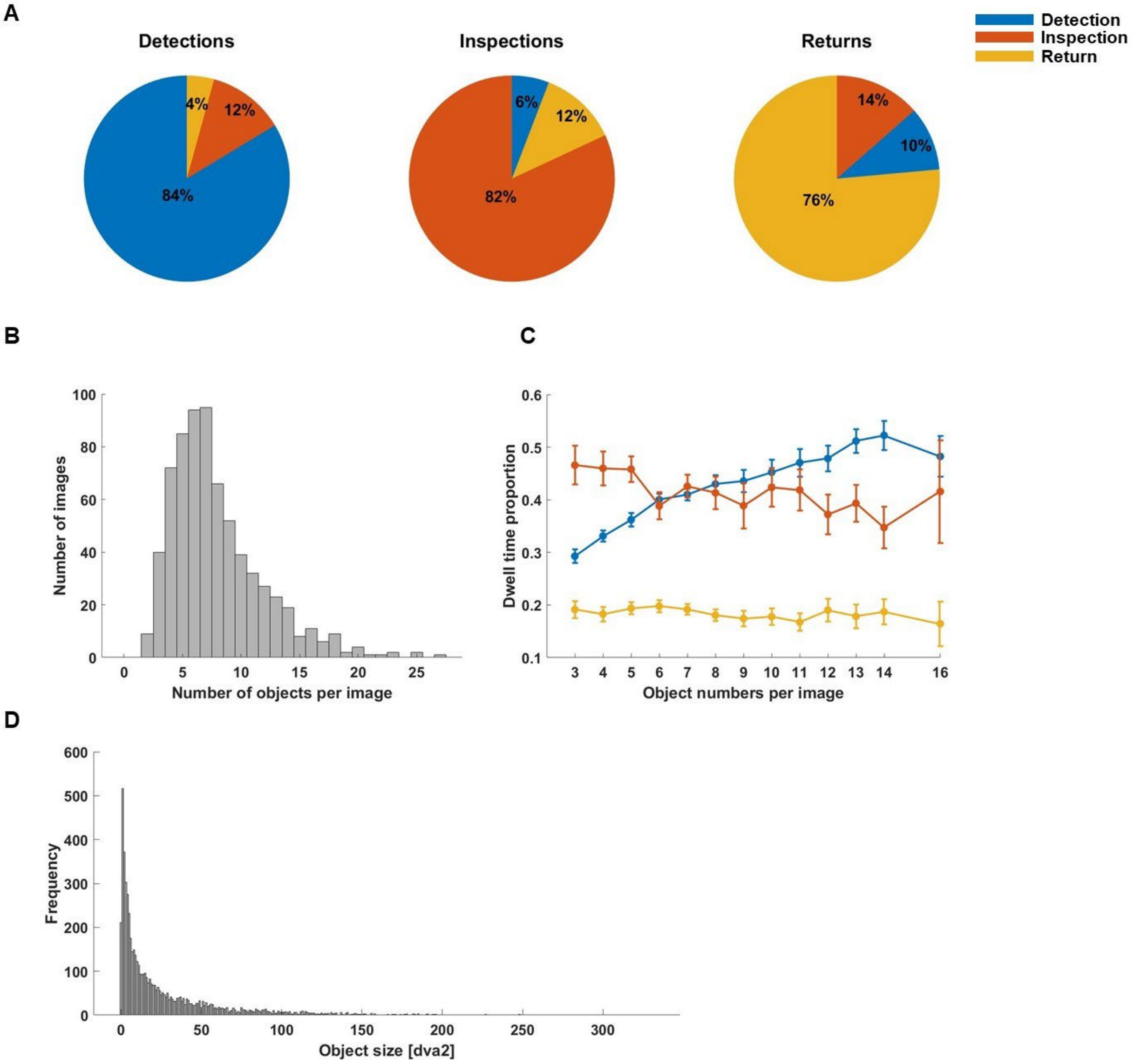

Figure S2. Descriptive analyses of fixation categories and object properties.
(A) Overlap of Detection, Inspection, and Return (D, I, R) labels across fixations. Each pie chart shows the proportion of fixations uniquely assigned to one category versus those also labelled as one or both of the other categories. (B) Histogram showing the number of annotated objects per image across the stimulus set. (C) Proportion of dwell time proportion spent on D, I, and R fixations as a function of the number of objects per image. Lines represent group means across images; error bars show 95% confidence intervals. (D) Distribution of object sizes across all annotated masks, shown in square degrees of visual angle (dva^2). Colours in (A) and (C) indicate fixation types as shown in the legend.

Fixation labelling without tolerance margin

To assess whether our findings are sensitive to the use of an error margin in fixation labelling, we repeated all main-text analyses without applying the 0.5 dva tolerance margin that was used in the original analyses. Overall, the results without a tolerance margin closely matched those from the main analysis. As illustrative examples, we present (1) D, I, and R dwell time proportions across semantic dimensions (Figure S3), and (2) fixation duration and inter-fixation distance over trial duration (Figure S4) - each shown side by side with and without the tolerance margin. In both cases, the patterns of results remain highly consistent.

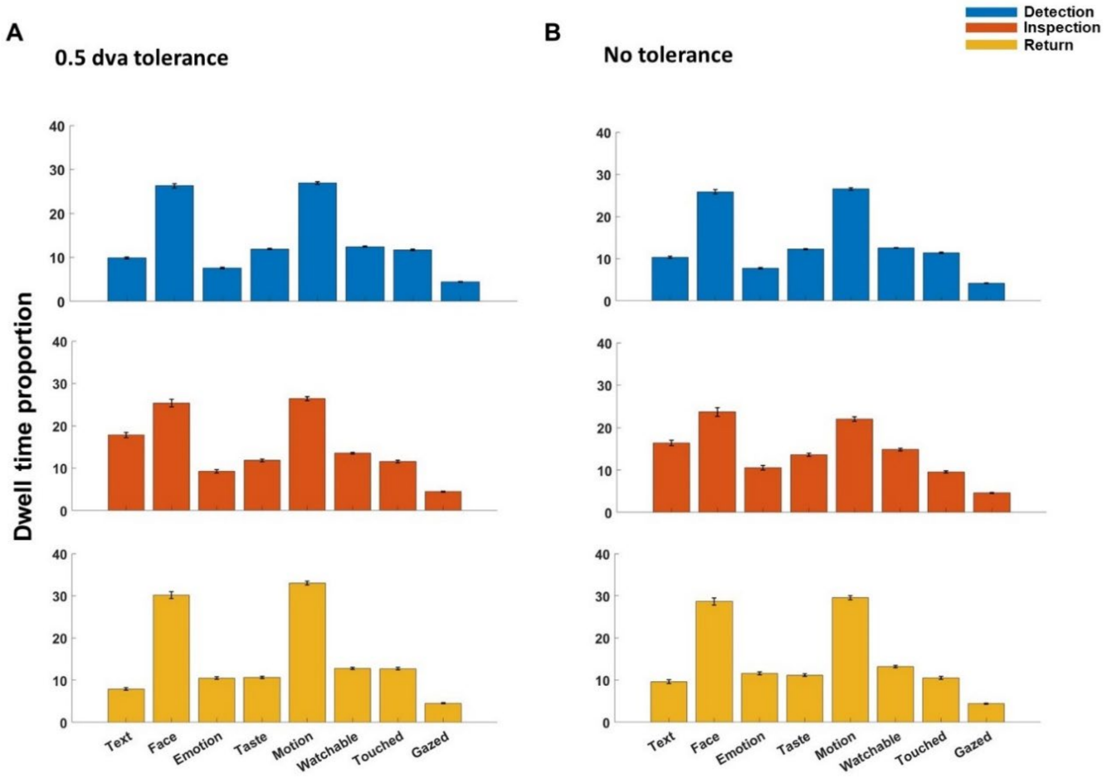

Figure S3. Dwell time proportion of D, I, and R fixations across semantic features labelled with and without applied error margin. Bar plots show the proportion of cumulative dwell time for Detection, Inspection, and Return fixations on objects of a given semantic feature, with (A) and without (B) a 0.5 dva tolerance margin applied during with fixation labelling. Fixation types are colour-coded as indicated in the inset. Error bars represent CI95% across observers.

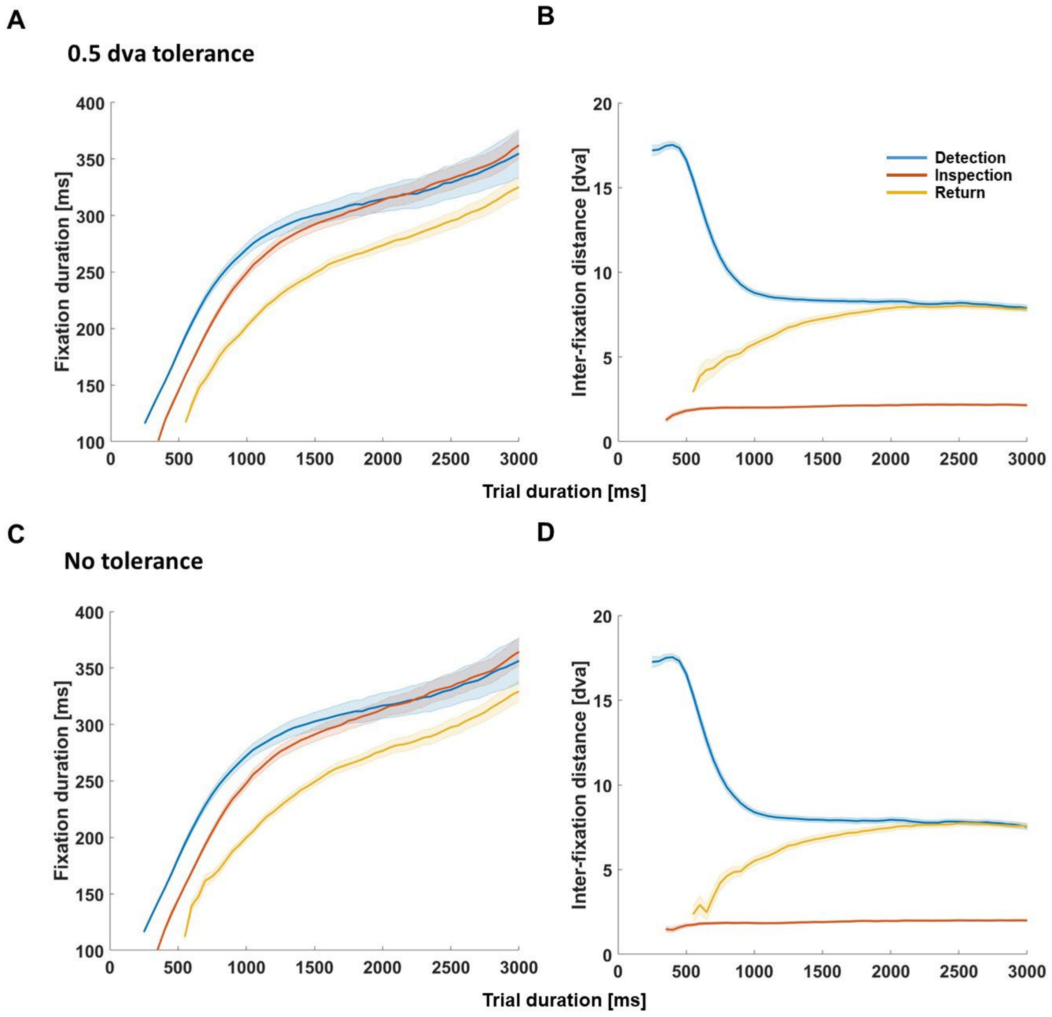

Figure S4. Fixation duration and inter-fixation distance over trial time with and without applied error margin. Line plots show the mean fixation duration (A, C) and mean Euclidean distance (in dva) to the previous fixation (B, D) for Detection, Inspection, and Return fixations across the full trial duration (150–3000 ms), calculated using 50 ms time bins. Panels A and B show results using a 0.5 dva tolerance margin for fixation labelling, while Panels C and D show results without applying a tolerance margin. Shaded areas represent 95% confidence intervals across observers. Fixation types are colour-coded as indicated in the inset.

However, the distribution and temporal dynamic of D, I, and R dwell time proportions slightly changed in the analysis without error margin. Figure S5 illustrates the time course (A and C) and the overall average dwell time proportions (B and D) of D, I, R, and unlabelled fixations, both with (A/B) and without (C/D) the applied error margin.

When no tolerance margin was used (C), the proportion of Inspection fixations still increased steeply early in the trial, plateauing around 900 ms, but did so at a noticeably lower level compared to the version with a tolerance margin (~0.35 vs. ~0.45). Additionally, the proportion of unlabelled fixations remained low in early time bins but reached a higher overall level by the end of

the trial. This shift is also reflected in the overall dwell time proportions (Panel D), showing a reduced proportion of Inspection fixations and a corresponding increase in unlabeled fixations when no margin was applied. This likely reflects small eye-tracking inaccuracies, registering (Inspection) fixations close to the edge of an object just outside of this object and underscores the usefulness of the error margin. Detection and Return fixations showed smaller differences between the two

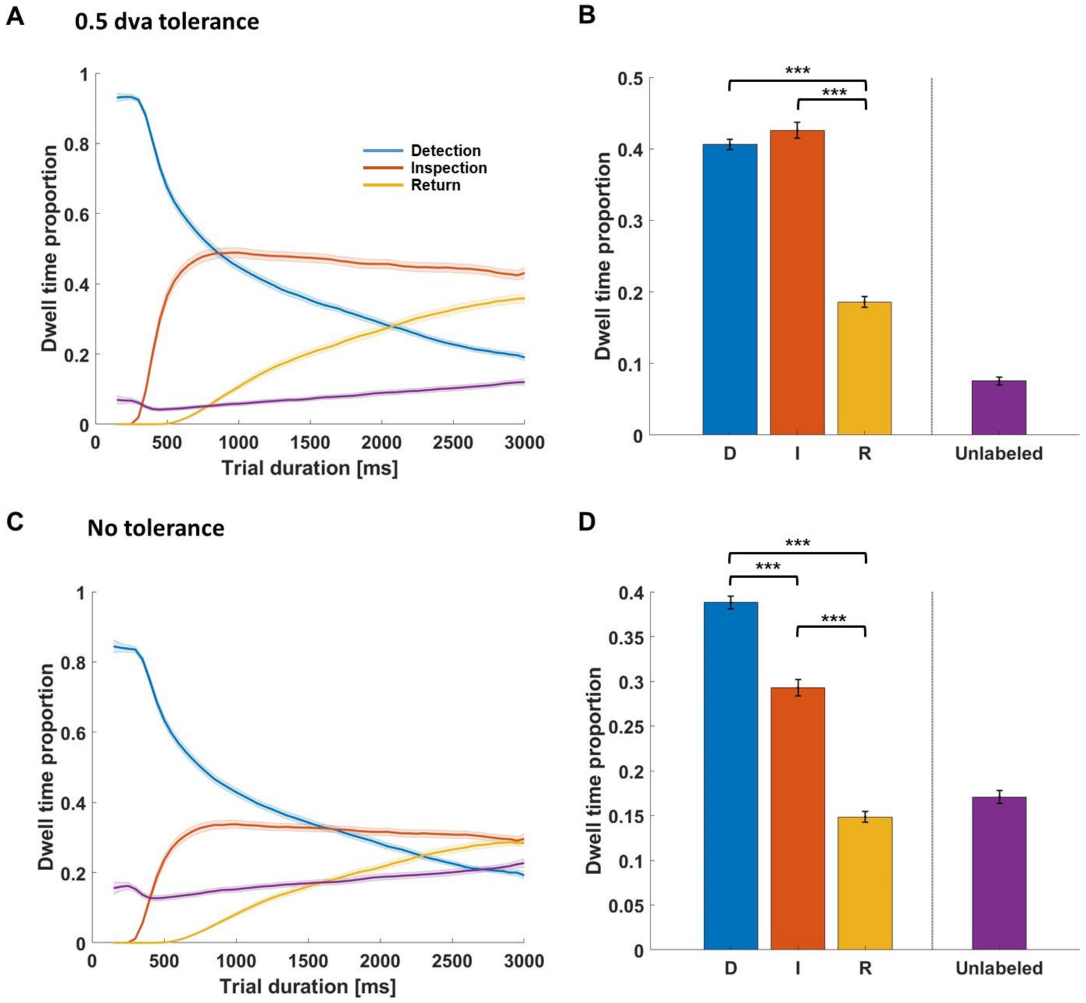

Figure S5. Fixation proportions across trial duration with and without applying error margin. Line plots depict the mean dwell time proportions of Detection, Inspection, and Return fixations across trial time (150–3000 ms), calculated in 50 ms bins. Panels A and B show results using a 0.5 dva tolerance margin when labelling fixations; Panels C and D show results without applying a tolerance margin. In Panels A and C, shaded areas represent 95% confidence intervals across observers. Panels B and D show the average cumulative dwell time proportions across the full 3-second viewing duration. Fixation types are colour-coded as indicated in the legend. Asterisks indicate significance levels: *$p < .05$, **$p < .01$, ***$p < .001$.

approaches.

Differential salience for D, I and R using Linear Mixed Effects Models

To examine potential differences in salience between Detection, Inspection, and Return fixations while accounting for individual differences in semantic salience, we modelled the relative dwell time allocated to semantic features across fixation types using a Linear Mixed-Effects model. The model revealed a Fixation type × Semantic feature interaction; $F(14, 2400) = 423.82$, $p < .001$).

Follow-up pairwise comparisons between fixation types within each semantic category revealed significant differences in dwell time allocation across most features (see Table 1). Specifically, all three fixation types (D, I, R) differed significantly in their dwell time proportions for *Face*, *Emotion*, *Motion* and *Text* (all $p < .01$). For *Touched*, significant differences were observed between Detection and Return ($p < .001$) and between Inspection and Return ($p < .001$), but not between Detection and Inspection ($p = .42$). For *Taste*, differences were significant between Detections and Return ($p < .001$) and Inspections and Returns ($p < .001$) , but not between Detection and Inspections ($p = .75$). For *Watchable*, dwell time proportion did not differ significantly between Detection and Return ($p = .06$), whereas the other pairs were significant ($p < .001$). For *Gazed*, dwell time did not differ significantly across D, I and R (all $p > .05$).

Table 1: Pairwise comparisons between Fixation types by Semantic feature

**Semantic feature**	**Fix. type 1**	**Fix. type 2**	***F* (1, 2400)**	***p***
Text	Detection	Inspection	2010.80	< .001
Text	Detection	Return	124.86	< .001
Text	Inspection	Return	3137.80	< .001
Face	Detection	Inspection	26.54	< .001
Face	Detection	Return	476.62	< .001
Face	Inspection	Return	728.10	< .001
Emotion	Detection	Inspection	92.44	< .001
Emotion	Detection	Return	269.45	< .001
Emotion	Inspection	Return	46.25	< .001
Taste	Detection	Inspection	0.10	.749

**Semantic feature**	**Fix. type 1**	**Fix. type 2**	**F (1, 2400)**	***p***
Taste	Detection	Return	51.80	< .001
Taste	Inspection	Return	47.30	< .001
Motion	Detection	Inspection	7.78	.005
Motion	Detection	Return	1192.30	< .001
Motion	Inspection	Return	1392.70	< .001
Watchable	Detection	Inspection	36.55	< .001
Watchable	Detection	Return	3.47	.063
Watchable	Inspection	Return	17.50	< .001
Touched	Detection	Inspection	0.66	.416
Touched	Detection	Return	33.57	< .001
Touched	Inspection	Return	43.65	< .001
Gazed	Detection	Inspection	0.05	.830
Gazed	Detection	Return	0.45	.501
Gazed	Inspection	Return	0.21	.646

Overall, these findings align with those retrieved using a RM ANOVA analyses (see main text) and confirm that attentional allocation across D, I and R fixations is not uniformly distributed across semantic features, with stronger differentiation for some features (e.g., *Face*, *Motion*, *Text*) and convergence for others (e.g., *Gazed*, *Taste*, *Touched*).
